# Moral Insanity

**Published:** 1882-05

**Authors:** 


### MORAL INSANITY.

This is a topic just now attracting much attention. Adopted as a definitive form of insanity by nearly all the English, French, German and Italian alienists, its existence is denied by a small circle of American superintendents, more in obedience to popular clamor than for any scientific reason. That this circle does not include all the superintendents is shown by the recent scientific article on the subject by Dr. Hughes (*Alienist and Neurologist*, January, 1882), who places himself on record with Ray and Rush, the Nestors of American psychiatry. Perhaps no one has condemned the position recently taken by certain alienist and non-alienist experts, that "moral insanity is unknown to medical science," in stronger or more deservedly severe terms than those used by Dr. Folsom. He says (*New York Medical Journal*, February, 1882): "The expert witness who denies that moral insanity exists, in the face of such careful and conclusive authorities as Dr. Ray and many others, declares that criminal acts are always criminal, that kleptomania is always stealing, dipsomania is always drunkenness and nothing more, is himself in danger of a certain unwholesome self-conceit that might itself be likened to moral insanity." Even the *Medical Record* has denounced the retrograde tendencies which have been recently shown in this particular respect.—*Chicago Medical Review.*

